# Memristive Switching and Density-Functional Theory Calculations in Double Nitride Insulating Layers

**DOI:** 10.3390/mi13091498

**Published:** 2022-09-09

**Authors:** Sobia Ali Khan, Fayyaz Hussain, Daewon Chung, Mehr Khalid Rahmani, Muhammd Ismail, Chandreswar Mahata, Yawar Abbas, Haider Abbas, Changhwan Choi, Alexey N. Mikhaylov, Sergey A. Shchanikov, Byung-Do Yang, Sungjun Kim

**Affiliations:** 1 A School of Electronics Engineering, Chungbuk National University, Cheongju 28644, Korea; 2 Materials Simulation Research Laboratory (MSRL), Department of Physics, Bahauddin Zakariya University, Multan 60800, Pakistan; 3 Division of Electronics and Electrical Engineering, Dongguk University, Seoul 04620, Korea; 4 Department of Physics, Khalifa University, Abu Dhabi 127788, United Arab Emirates; 5 School of Electrical and Electronic Engineering, Nanyang Technological University, Singapore 639798, Singapore; 6 Division of Materials Science and Engineering, Hanyang University, Seoul 04763, Korea; 7 Research and Education Center “Physics of Solid State Nanostructures”, National Research Lobachevsky State University of Nizhny Novgorod, 603022 Nizhny Novgorod, Russia; 8 Department of Information Technologies, Vladimir State University, 600000 Vladimir, Russia

**Keywords:** resistive switching, silicon nitride, boron nitride, self-rectification

## Abstract

In this paper, we demonstrate a device using a Ni/SiN/BN/p^+^-Si structure with improved performance in terms of a good ON/OFF ratio, excellent stability, and low power consumption when compared with single-layer Ni/SiN/p^+^-Si and Ni/BN/p^+^-Si devices. Its switching mechanism can be explained by trapping and de-trapping via nitride-related vacancies. We also reveal how higher nonlinearity and rectification ratio in a bilayer device is beneficial for enlarging the read margin in a cross-point array structure. In addition, we conduct a theoretical investigation for the interface charge accumulation/depletion in the SiN/BN layers that are responsible for defect creation at the interface and how this accounts for the improved switching characteristics.

## 1. Introduction

Advanced memristor-type non-volatile memory technology such as magneto-resistive memory (MRAM), phase-change memory (PRAM), and resistive memory (RRAM) are encouraging researchers to pursue better device performance than what is available from traditional Si-based flash memory [1]. RRAM is considered to be one of the most promising devices for next-generation memory applications due to its high density, low-power consumption, high speed, and good compatibility with conventional semiconductor processes [2,3,4]. The unipolar and bipolar switching of RRAM devices carries out reversible low resistive switching (LRS) and high resistive switching (HRS) depending on the bias polarity and amplitude. RRAM devices can be switched from HRS to LRS in the set process and then from LRS to HRS in the reset process after the electroforming process; however, some devices also have forming-free behavior [5]. RRAM can be employed as a semiconductor memory chip for use as storage-class memory (SCM) and embedded non-volatile memory depending on the performance type that is determined by the various materials used. A simple RRAM cell is easily integrated with a 3D vertical structure arranged in a cross-point manner. When using RRAM as storage memory, the 3D stacking density becomes an important factor [6]. For PRAM and RRAM, a unit cell area of 4F^2^ (F is feature size) can be achieved because the cells are arranged in a cross-point array structure [7]. However, in the cross-point structure, when reading a specific cell, there are sneak current paths through adjacent cells. These paths reduce the read margin and may cause array size to be limited. This sneak current path can be minimized in a cross-point array when a nonlinear, rectifying current voltage is achieved. To maintain a unit cell area of 4F^2^, a capacitor-like stack on the memory cell is essential. In contrast, the integration of transistors would lead to a much larger unit area (>6F^2^). Ovonic threshold switches (OTS) based on chalcogenide materials are used mainly as selection devices in 3D Xpoint memory. The fabrication process for OTS is temperature sensitive and requires a barrier layer between the memory device and the selector layer to reduce intermixing [8].

RRAM is composed of a metal-insulator-silicon (MIS) structure and is more flexible in terms of nonlinear and self-rectifying properties by work function adjustment in the bottom electrode (BE) silicon surface compared to traditional devices [9]. Among the many nitride-based resistive materials, SiN is considered the best switching material due to the abundant traps in the insulator [9]. Furthermore, SiN-based RRAM has numerous other advantages such as low switching current, good endurance, strong retention, fast switching speed, and fully compatible with conventional Si CMOS processing [10,11,12,13,14,15]. The conduction mechanism of SiN-based RRAM can be explained by the electron trapping and de-trapping procedures that take place due to the dangling bonds of Si; these processes are governed by the trap-controlled space-charge-limited current (SCLC) model [16]. Boron nitride (BN) is known to be thermally stable, electrically insulating and chemically passive [17,18]. BN is normally found in a crystalline form with various structures, i.e., hexagonal (h-BN), cubic (c-BN) and Wurtzite (w-BN); however, it can also be found as amorphous (a-BN), which is a non-crystalline form with long distance irregularity in the arrangement of the atoms [18]. Thin films of a-BN are transparent and insulating so are considered a great dielectric for use in electronic devices, on top of this they can be deposited with the most commonly used chemical vapor deposition (CVD) process.

In this work, we use amorphous boron nitride in the resistive switching layer of our bipolar resistive switching device to achieve a better device performance in comparison to that of the traditional Si-based RRAM [19,20,21]. Herein, we report on a Ni/SiN/BN/p^+^-Si device with non-linearity and self-rectifying characteristics. We demonstrate the device’s good nonlinear behavior that comes from its double nitride layer by calculating (for each layer) its forward to reverse (F/R) current ratio, selectivity, and read margin and solve the sneak path current issue. To prove that the electrode limited conduction mechanism has a linear slope for Schottky emission, a curve is fitted to the measured I-V data with temperature dependence being considered.

## 2. Experiments

Ni/SiN/BN/p^+^-Si devices were fabricated as follows: the p-type dopant (BF_2_^+^) as BE was implanted into the silicon surface with an acceleration energy of 40 keV and a dose of 5 × 10^13^ cm^−2^. The annealing was carried out at 1050 °C for 10 min to restore the silicon lattice damaged during ion-implantation. A 4 nm-thick BN was deposited by RF magnetron sputtering using a ceramic target of boron nitride on a highly doped silicon substrate at room temperature. Subsequently, 4 nm-thick SiN was deposited by plasma-enhanced chemical vapor deposition (PECVD) at about 300 °C using 5% SiH_4_/N_2_ (800 sccm), NH_3_ (10 sccm), and N_2_ (1200 sccm) on a p^+^-Si substrate. For top electrode (TE) deposition, DC magnetron sputtering was used to deposit 100 nm thick Ni top electrodes with a diameter of 100 μm. All direct current (DC) voltage sweep electrical properties were recorded using a Keithley 4200-SCS (Cleveland, OH, USA) and Keysight B1500A (Santa Rosa, CA, USA) semiconductor parameter analyzer. For all device measurements, the Si bottom electrodes were grounded, and the voltage bias was applied to the Ni electrode.

Based on density functional theory, theoretical confirmation of conducting filaments formation, geometry optimizations, and electronic density of states (DOS) calculations were performed using the generalized gradient approximation (GGA) along with Perdew, Burke, and Ernzerhof (PBE) functionals and the help of the Vienna Ab-initio Simulation Package (VASP) [22,23,24]. First, the amorphous SiN and BN supercell were modeled using the molecular dynamics package in a Large-scale Atomic/Molecular Massively Parallel Simulator (LAMMPS) [25], then prepared the (001) surface of both samples so that they were close to our experimental example. Convergence tests for the system’s total energy with respect to electron wave functions were conducted using plane waves with cut-off energy of 500 eV. The ionic positions, cell volume, and system lattice parameters were fully relaxed using the conjugate gradient (CG) method until the Hellmann Feynman forces became smaller than 0.02 eV/Å and the energy convergence criteria were met at 1 × 10^−5^ eV [26]. The most popular scheme for these calculations is Monkhorst Pack (MP) [27,28]. This was applied for k-point sampling. The MP grid was chosen to be 10 × 10 × 8.

## 3. Results and Discussions

**Figure 1:**
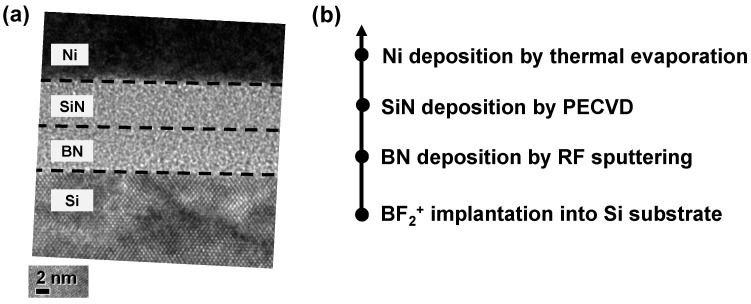
(**a**) TEM image and (**b**) process flow of Ni/SiN/BN/p^+^-Si device.

Figure 1a presents the transmission electron microscopy (TEM) image of the double layer device with a Ni/SiN/BN/p^+^-Si stack. The SiN and BN layers are each approximately 4 nm thick, so the total thickness of the two dielectrics together is about 8 nm. The amorphous SiN and BN layers and the single crystalline silicon substrate can be clearly observed. Figure 1b shows the process flow we discussed in the method section.

It is difficult to clearly distinguish the two nitride layers, but about a 4 nm-thick BN single layer was deposited and confirmed by another TEM image which is not shown here. We confirmed the material and chemical composition of the SiN layer and BN layer in our previous works [29,30].

**Figure 2:**
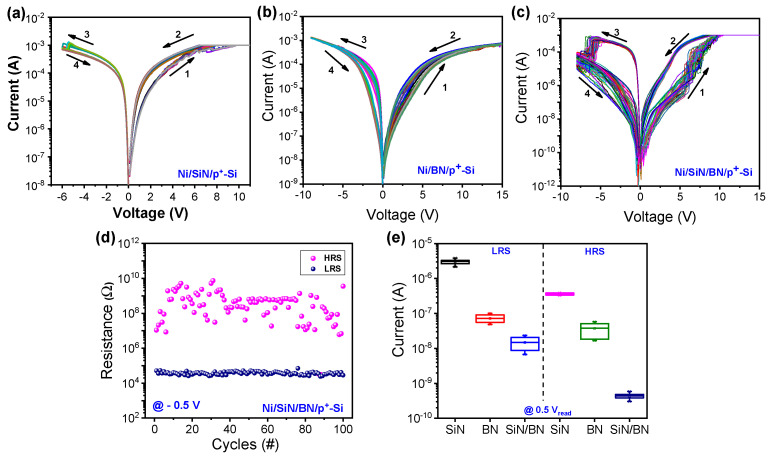
Typical I-V characteristics of (**a**) Ni/SiN/p^+^-Si, (**b**) Ni/BN/p^+^-Si, and (**c**) Ni/SiN/BN/p^+^-Si devices. (**d**) Endurance of Ni/SiN/BN/p^+^-Si device. (**e**) LRS and HRS distribution of three devices.

Figure 2a–c shows the current-voltage (I-V) characteristics of the Ni/SiN/p^+^-Si, Ni/BN/p^+^-Si, and Ni/SiN/BN/p^+^-Si devices, respectively. The set process occurs with current compliance (CC) of 1 mA to achieve the low-resistance state (LRS). The device switches from LRS to a high-resistance state (HRS) with a negative polarity sweep. This switching behavior is related to the traps formed in the SiN film and this is further affected by the deposition method of the SiN film, as shown in Figure 2a. The conduction mechanism can be explained by the space-charge-limited current (SCLC) model and the accompanying electron trapping and de-trapping in the SiN film [16]. When electrons are trapped in the trap site, the conductivity increases; conversely, when a de-trap occurs, the conductivity decreases. The device shows gradual switching during the set process and nonlinear LRS behavior. The set transition starts at about 5 V. During the reset process, the device changes from LRS to HRS, indicating filament rupture. The reset process occurs at about −4.5 V, but a small current change is observed. Similar resistive switching properties are observed in the Ni/BN/p^+^-Si device in Figure 2b. However, the on/off ratio reduces with increasing number of switching cycles. So, few cycles of endurance are recorded. The Ni/SiN/BN/p^+^-Si device shows a better on/off ratio, which is important for non-volatile memory in Figure 2c. The set and reset transitions occur at about 8 V and −6 V, respectively.

Figure 2d shows the endurance of the Ni/SiN/BN/p^+^-Si device. The LRS becomes more stable and shows good uniformity over 100 consecutive cycles of DC sweep, but the HRS resistance fluctuates due to a multitude of different ways in which filament rupture may occur. There are enough boron and nitride vacancies present for confined conducting filaments to act as growth modulators of CFs that are related to their shape and location. This may be caused by the formation and rupture of filaments occurring in the same location. The endurance could be improved by additional approaches such as thickness and defect optimization. Figure 2e shows the HRS and LRS distributions of three devices. The bilayer device shows lower current levels in both LRS and HRS.

**Figure 3:**
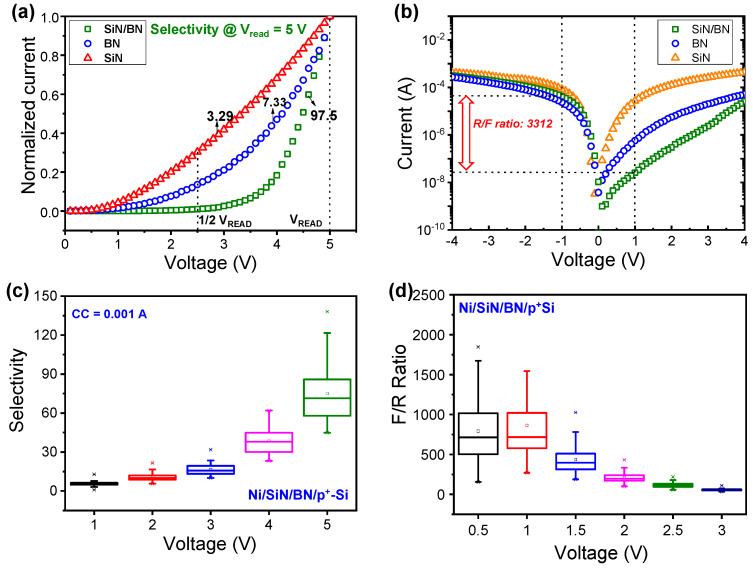
Selectivity and F/R ratio of Ni/SiN/p^+^-Si, Ni/BN/p^+^-Si, and Ni/SiN/BN/p^+^-Si devices in LRS: (**a**) normalized current curves of selectivity and (**b**) I-V curves for F/R ratio. (**c**) Selectivity and (**d**) F/R ratio as a function of read voltage.

The SiN/BN device has an advantage in that the LRS current is significantly suppressed and nonlinear during positive bias. In Figure 3a, we compare selectivity, defined as the ratio of the current at V_READ_ to that at 0.5V_READ_, using the normalized current of the Ni/SiN/p^+^-Si, Ni/BN/p^+^-Si, and Ni/SiN/BN/p^+^-Si devices. Figure 3b shows the LRS current of the Ni/SiN/p^+^-Si, Ni/BN/p^+^-Si, and Ni/SiN/BN/p^+^-Si devices to compare the forward current/reverse current (F/R ratio) between them. The Ni/SiN/BN/p^+^-Si device shows the highest F/R ratio due to reverse current suppression. In contrast to selectivity, the highest F/R ratio is achieved at a low-read voltage of 1 V due to the current suppression that occurs during a low voltage regime with a positive bias. The Ni/SiN/BN/p^+^-Si device shows the highest selectivity among the three devices. The selectivity increases with increasing read voltage, as shown in Figure 3c. Figure 3d shows a box chart of the F/R ratio as a function of the read voltage for the SiN/BN device. Selectivity and F/R ratio show opposite trends as read voltage increases; as such, the read voltage should be carefully selected to give a large read margin.

**Figure 4:**
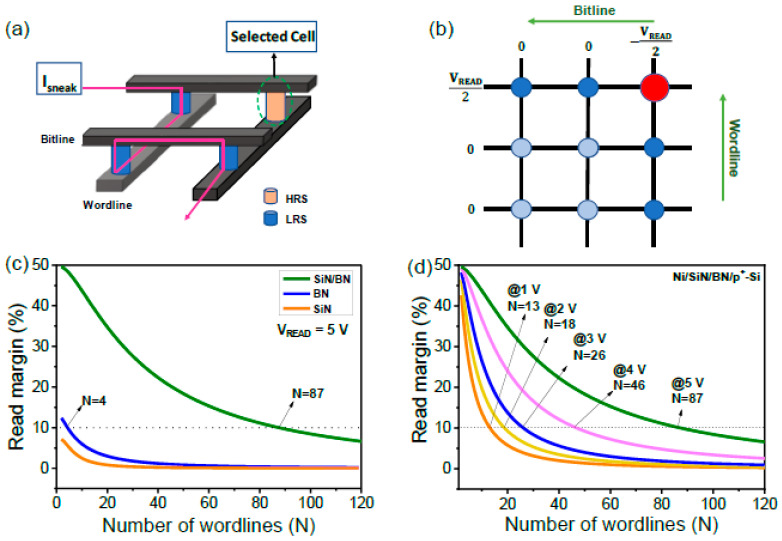
(**a**) Schematic of cross-point array illustrating sneak current. (**b**) Half-bias read scheme of cross-point structure. (**c**) Read margin with number of word lines of Ni/SiN/p^+^-Si, Ni/BN/p^+^-Si, and Ni/SiN/BN/p^+^-Si devices. (**d**) Read margin with number of word lines of Ni/SiN/BN/p^+^-Si device at different read voltages (1 V, 2 V, 3 V, 4 V, and 5 V).

Given the nonlinear and self-rectifying manners seen in LRS, bipolar resistive switching can alleviate sneak paths through the LRS elements in a cross-point array. Memory cells are located at the intersection of bit lines and word lines in a cross-point array, as shown in Figure 4a. When we select a cell for reading, the current flow through unselected cells can cause readout errors and crosstalk problems. In a cross-point array, the cross-talk between adjacent memory cells restricts the maximum array size [31]. The half-bias read scheme estimates the cross-point array size for the three devices in Figure 4b. *V_READ_* is applied to the selected cell and half-bias or zero bias is applied to the memory cells. The read margin as a function of array size can be calculated by output voltage across pull-up resistance (*R_pu_*) and solving the Kirchhoff equation given the equation below [32].

(1) $$\frac{\Delta V}{V_{pu}}\left(N\right)=\frac{R_{pu}}{\left(R_{LRS}\left(V_{READ}\right)||\left(\frac{2R_{LRS}\left(\frac{V_{READ}}{2}\right)}{N-1}+\frac{R_{LRS}\left(\frac{V_{READ}}{2}\right)}{{\left(N-1\right)}^{2}}\right)\right)+R_{pu}}-\frac{R_{pu}}{\left(R_{HRS}\left(V_{READ}\right)||\left(\frac{2R_{LRS}\left(\frac{V_{READ}}{2}\right)}{N-1}+\frac{R_{LRS}\left(\frac{V_{READ}}{2}\right)}{{\left(N-1\right)}^{2}}\right)\right)+R_{pu}}$$

Figure 4c shows the calculated read margin for all three RRAM devices as a function of word line quantity at a *V_READ_* of 5 V. Ni/SiN/p^+^-Si and Ni/BN/p^+^-Si devices show a sharp read margin decrease as the array size increases. In contrast, the read margin of a SiN/BN device decreases much more slowly as the array size increases; this is due to its higher on/off ratio, selectivity and R/F ratio. We also compared read margins as a function of the number of word lines using different voltages for the Ni/SiN/BN/p^+^-Si device, as shown in Figure 4d. The number of word lines for a read margin of at least 10% is 13, 18, 26, 46 and 87 at read voltages of 1 V, 2 V, 3 V, 4 V, and 5 V, respectively. The selectivity is more important than the R/F ratio in a typical half-read scheme. A high read voltage is not favorable for low-power operation.

**Figure 5:**
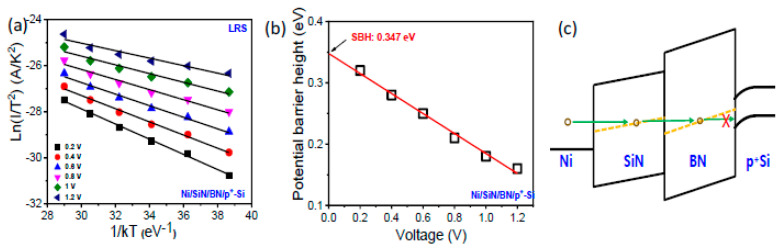
(**a**) ln(I/T^2^) versus 1/KT plot. (**b**) Effective barrier height as a function of voltage for Ni/SiN/BN/p^+^-Si in LRS under negative bias. (**c**) Energy band diagram of Ni/SiN/BN/p^+^-Si device.

To analyze the suppression of reverse current, we conducted temperature characteristic I-V fitting. Figure 5a shows an ln (I/*T*^2^) versus 1/kT plot for the Ni/SiN/BN/p^+^-Si device under reverse LRS bias for Schottky emission with the following expression [33,34].

(2) $$J=A^{*}\;T^{2}\;\exp\left(-\frac{q\;(\Phi_{B}-\sqrt{qE/4\pi \varepsilon_{r}\varepsilon_{0}}\;}{k_{B}T}\right)$$

where *J* is the current density, A* is the effective Richardson constant, *T* is absolute temperature, *q* is the electron charge, Φ*_B_* is effective barrier height, *E* is the electric field, *ε_r_* is dynamic dielectric constant, *ε*_0_ is the dielectric constant of free space, and *k_B_* is Boltzmann constant. Each voltage barrier height can be extracted from the slope of the ln (I/*T*^2^) versus 1/kT plot in Figure 5a,b. In LRS, many traps can be generated around the midgap of the dielectrics when conducting defects are induced in the SiN and BN layers by the set process. This allows carriers to move through the trap, as shown in Figure 5c [15]. When a positive bias is applied to a SiN/BN device, the holes from Ni TE can tunnel into the SiN and BN layer trap state and then be blocked by a Schottky barrier in the p^+^-Si.

**Figure 6:**
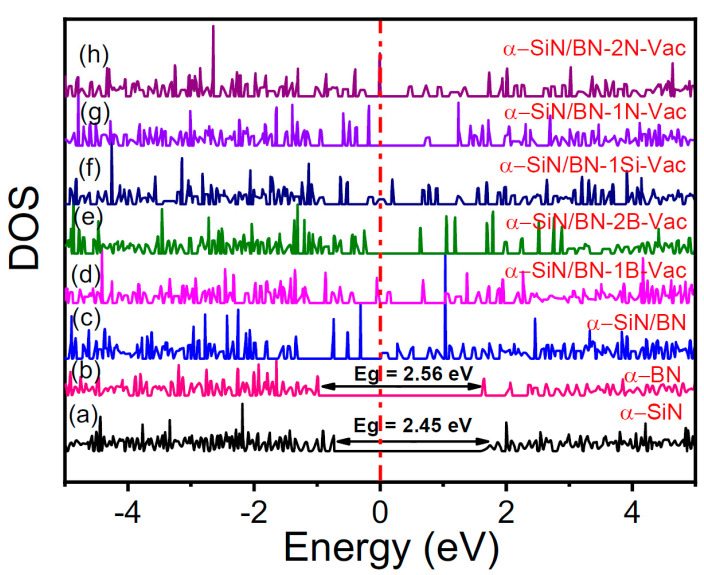
Density of states (DOS): (**a**) α-SiN, (**b**) α-BN, (**c**) α-SiN/BN, (**d**) α-SiN/BN-1B-Vac (Vacuum), (**e**) α-SiN/BN-2B-Vac, (**f**) α-SiN/BN-1Si-Vac, (**g**) α-SiN/BN-1N-Vac, (**h**) α-SiN/BN-2N-Vac. (N is effective density of function).

**Figure 7:**
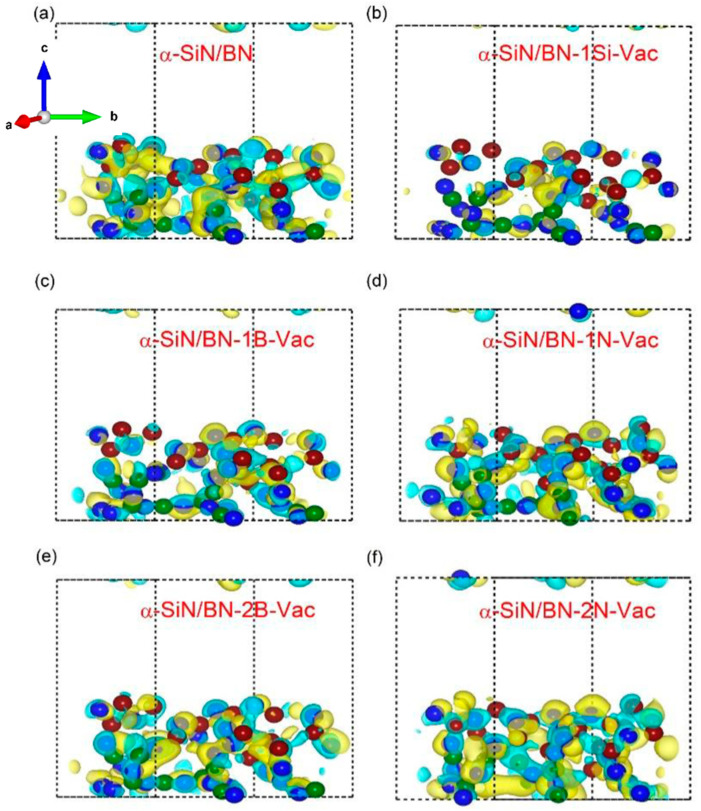
Iso-surface charge density. a, b, c axis means x, y, z axis of the device: (**a**) α-SiN/BN, (**b**) α-SiN/BN-1Si-Vac, (**c**) α-SiN/BN-1B-Vac, (**d**) α-SiN/BN-1N-Vac, (**e**) α-SiN/BN-2B-Vac, (**f**) α-SiN/BN-2N-Vac.

The density of states (DOS) and iso-surface charge density plots of SiN and BN with a single silicon vacancy, a nitrogen vacancy as well as double nitrogen/boron vacancies are used to investigate the charge transformation phenomena, as shown in Figure 6a–h. The calculated bandgap values for SiN and BN are 2.46 eV and 2.57 eV, respectively, which are close to the values reported in the literature (2.0 eV for BN) [35], but less than previous experimental values of 3.5 eV and 5.0 eV, respectively [36,37]. The underestimation in the bandgap calculation is caused by the exchange and correlation GGA function between electrons and does not affect the analysis of the system used [38]. Expectations related to sharing the 3p-electrons of Si and 2p-electrons of N are that p-p hybridization occurs in a similar way to how the 2p-electrons of B and 2p-electrons of N also follow p-p hybridization. Therefore, an interface layer may be formed (Si-N-B) between SiN and BN that has the ability to enhance conductivity by generating conducting defects at the interface as well as at interstitial locations between atoms. It is expected that Si/B can extract nitrogen from the SiN/BN bilayer to form a Si-N-B charge layer. The formation and rupture of conductive filaments, probably due to nitrogen and boron vacancies, are represented at the resistive switching of the SiN/BN bilayer in Figure 6c. This allows for charges/electron movement between interface layers during the switching phenomena caused by defect states, as represented by the Fermi level in Figure 6d,e. Numerous charges have accumulated at the interface as well as at the inter-sites between Si-N-B atoms and near nitrogen/boron vacancies, represented by the yellow color in the iso-surface charge density plots from Figure 7a–f.

DFT layers are vertically stacked along the *z*-axis, and more commonly, the c-axis. In contrast, iso-surface charge density plots show the qualitative nature of conducting filaments formed along with the optimized structure or at the interface. The large numbers of filamentary channel formation for interface type switching are also responsible for high conductivity. Figure 7c,e,f elaborate on, and the Bader charge analysis confirms, the results in Table 1. The locations where numerous charges accumulate/are depleted (−2.13, −1.0 and +2.11, +2.85) are represented with yellow and cyan colors, respectively, for single and double nitrogen as well as boron vacancies in the Si/BN composite structure [39].

## 4. Conclusions

We demonstrated self-selective resistive switching in a Ni/SiN/BN/p^+^-Si device. This bilayer device shows an improved on/off ratio and stability over up to 100 consecutive cycles. A Schottky barrier at the silicon surface can significantly suppress the reverse current. The nonlinear and self-rectifying characteristics of double nitride layer devices in LRS are beneficial to high-density memory implementation without a selector device due to the fact they help to alleviate sneak current paths in a cross-point array. It was found that both pure and defected nitrogen/boron vacancies are formed and ruptured; this allows numerous electronic states at the Fermi level, which can be seen in the iso-surface charge density of the SiN/BN bilayer device.

## Figures and Tables

**Table 1:** Bader charge analysis of nearest neighboring atoms (Si, B, N).

	Ni/SiN/ BN/Si	1 B Vac	1 Si Vac	1 N Vac	2 B Vac	2 N Vac
Si	+2.85	+2.91	+2.81	+3	+0.85	+1.04
B	+2.11	+2.1	+2.14	+2.1	+2.13	+2.14
N	−0.8	−1	−0.86	−2.13	−0.71	−2.13

## Data Availability

Not applicable.

## References

[1] Yang J.J., Strukov D.B., Stewart D.R. (2013). Memristive devices for computing. Nat. Nanotechnol..

[2] Banerjee W., Xu X., Lv H., Liu Q., Long S., Liu M. (2017). Complementary switching in 3D resistive memory array. Adv. Electron. Mater..

[3] Gaba S., Cai F., Zhou J., Lu W.D. (2014). Ultralow sub-1-nA operating current resistive memory with intrinsic non-linear characteristics. IEEE Electron. Device Lett..

[4] Fang Y., Yu Z., Wang Z., Zhang T., Yang Y., Cai Y., Huang R. (2018). Improvement of HfO_X_-Based RRAM Device Variation by Inserting ALD TiN Buffer Layer. IEEE Electron. Device Lett..

[5] Pan T.M., Lu C.H. (2011). Forming-free resistive switching behavior in Nd_2_O_3_, Dy_2_O_3_, and Er_2_O_3_ films fabricated in full room temperature. Appl. Phys. Lett..

[6] Yu M., Cai Y., Wang Z., Fang Y., Liu Y., Yu Z., Pan Y., Zhang Z., Tan J., Yang X. (2016). Novel vertical 3D structure of TaO_X_-based RRAM with self-localized switching region by sidewall electrode oxidation. Sci. Rep..

[7] Yu S., Chen H.-Y., Gao B., Kang J., Wong H.-S.P. (2013). HfO_X_-Based Vertical Resistive Switching Random Access Memory Suitable for Bit-Cost-Effective Three-Dimensional Cross-Point Architecture. ACS Nano.

[8] Raty J.Y., Noé P. (2020). Ovonic Threshold Switching in Se-Rich Ge_x_Se_1−x_ Glasses from an Atomistic Point of View: The Crucial Role of the Metavalent Bonding Mechanism. Phys. Status Solidi RRL.

[9] Tikhov S.V., Mikhaylov A.N., Belov A.I., Korolev D.S., Antonov I.N., Karzanov V.V., Gorshkov O.N., Tetelbaum D.I., Karakolis P., Dimitrakis P. (2018). Role of highly doped Si substrate in bipolar resistive switching of silicon nitride MIS-capacitors. Microelectron. Eng..

[10] Kim S., Kim H., Hwang S., Kim M.-H., Chang Y.-F., Park B.-G. (2017). Analog Synaptic Behavior of a Silicon Nitride Memristor. ACS Appl. Mater. Interfaces.

[11] Kim S., Jung S., Kim M.H., Cho S., Park B.G. (2015). Resistive switching characteristics of Si_3_N_4_ -based resistive-switching random-access memory cell with tunnel barrier for high density integration and low-power applications. Appl. Phys. Lett..

[12] Kim S., Jung S., Kim M.-H., Chen Y.-C., Chang T.-C., Ryoo K.-C., Cho S., Lee J.-H., Park B.-G. (2018). Scaling Effect on Silicon Nitride Memristor with Highly Doped Si Substrate. Small.

[13] Kim H.D., Yun M.J., Kim S. (2016). Resistive switching characteristics of Al/Si_3_N_4_/p-Si MIS-based resistive switching memory devices. J. Korean Phys. Soc..

[14] Kim H.D., Yun M., Kim S. (2015). Self-rectifying resistive switching behavior observed in Si_3_N_4_ -based resistive random access memory devices. J. Alloy. Compd..

[15] Jiang X., Ma Z., Xu J., Chen K., Xu L., Li W., Huang X., Feng D. (2015). a-SiN_X_: H-based ultra-low power resistive random access memory with tunable Si dangling bond conduction paths. Sci. Rep..

[16] Hong S.M., Kim H.D., An H.M., Kim T.G. (2013). Effect of Work Function Difference Between Top and Bottom Electrodes on the Resistive Switching Properties of SiN Films. IEEE Electron. Device Lett..

[17] Zedlitz R., Heintze M., Schubert M.B. (1996). Properties of amorphous boron nitride thin films. J. Non-Cryst. Solids.

[18] Rand M.J., Roberts J. (1968). Preparation and Properties of Thin Film Boron Nitride. J. Electrochem. Soc..

[19] Kim D., Shin J., Kim S. (2021). Resistive Switching Characteristics of ZnO-Based RRAM on Silicon Substrate. Metals.

[20] Ryu H., Kim S. (2021). Effects of Oxygen Precursor on Resistive Switching Properties of CMOS Compatible HfO2-Based RRAM. Metals.

[21] Cho H., Kim S. (2020). Short-Term Memory Dynamics of TiN/Ti/TiO2/SiOx/Si Resistive Random Access Memory. Nanomaterials.

[22] Perdew J.P., Burke K., Ernzerhof M. (1996). Generalized Gradient Approximation Made Simple. Phys. Rev. Lett..

[23] Blöchl P.E. (1994). Projector augmented-wave method. Phys. Rev. B Condens. Matter Mater. Phys..

[24] Kresse G., Joubert D. (1999). From ultrasoft pseudopotentials to the projector augmented-wave method. Phys. Rev. B Condens. Matter Mater. Phys..

[25] Plimpton S. (1995). Fast parallel algorithms for short-range molecular dynamics. J. Comput. Phys..

[26] Krukau A.V., Vydrov O.A., Izmaylov A.F., Scuseria G.E. (2006). Influence of the exchange screening parameter on the performance of screened hybrid functionals. J. Chem. Phys..

[27] Monkhorst H.J., Pack J.D. (1976). Special points for brillouin-zone integrations. Phys. Rev. B Condens. Matter.

[28] Pack J.D., Monkhorst H.J. (1977). “Special points for Brillouin-zone integrations”-A reply. Phys. Rev. B Condens. Matter.

[29] Choi J., Kim S. (2020). Coexistence of Long-Term Memory and Short-Term Memory in an SiN_X_-Based Memristor. Phys. Status Solidi RRL.

[30] Lee J., Ryu J.-H., Kim B., Hussain F., Mahata C., Sim E., Ismail M., Abbas Y., Abbas H., Lee D.K. (2020). Synaptic Characteristics of Amorphous Boron Nitride-Based Memristors on a Highly Doped Silicon Substrate for Neuromorphic Engineering. ACS Appl. Mater. Interfaces.

[31] Linn E., Rosezin R., Kügeler C., Waser R. (2010). Complementary resistive switches for passive nanocrossbar memories. Nat. Mater..

[32] Aluguri R., Kumar D., Simanjuntak F.M., Tseng T.Y. (2017). One bipolar transistor selector—One resistive random access memory device for cross bar memory array. AIP Adv..

[33] Cowley A.M., Sze S.M. (1965). Surface states and barrier height of metal-semiconductor systems. J. Appl. Phys..

[34] Cheung S.K., Cheung N.W. (1986). Extraction of Schottky diode parameters from forward current-voltage characteristics. Appl. Phys. Lett..

[35] Durandurdu M. (2015). Hexagonal nanosheets in amorphous BN: A first principles study. J. Non-Cryst. Solids.

[36] Zanatta A.R., Nunes L.A.D.O. (1998). Green photoluminescence from Er-containing amorphous SiN thin films. Appl. Phys. Lett..

[37] Mary J.A., Vijaya J.J., Dai J.H., Bououdina M., John Kennedy L., Song Y. (2015). Experimental and first-principles DFT studies of electronic, optical and magnetic properties of cerium-manganese codoped zinc oxide nanostructures. Mater. Sci. Semicond. Process..

[38] Henkelman G., Arnaldsson A., Jónsson H. (2006). A fast and robust algorithm for Bader decomposition of charge density. Comput. Mater. Sci..

[39] Tang W., Sanville E., Henkelman G. (2009). A grid-based Bader analysis algorithm without lattice bias. J. Phys. Condens. Matter.

